# Lineage-specific selection signals in the Growth arrest-specific protein 8 (GAS8) domain protein of *Trypanosoma melophagium*

**DOI:** 10.1038/s41598-026-56233-x

**Published:** 2026-06-09

**Authors:** Anumita Das, Arnab Ghosh, Swarnamoy Pal, Sonali Biswas, Krishnendu Sinha, Debjani Sarkar, Nabanita Ghosh

**Affiliations:** 1 Department of Zoology, Maulana Azad College, Kolkata, 700013 India; 2 Department of Zoology, Jhargram Raj College, Jhargram, 721507 India

**Keywords:** GAS8 domain, Orthogroup 6127at5690, Cytoskeleton, Flagellar motility, Episodic selection, *Trypanosoma*, Evolution, Genetics

## Abstract

The Growth arrest-specific protein 8 (GAS8) domain-containing gene family (Orthogroup 6127at5690) is a highly conserved, single-copy orthologous group in the *Trypanosoma* taxa retained for codon-based evolutionary analysis (10 validated species). This family encodes a microtubule-associated protein implicated in cytoskeletal organization and flagellar motility. We conducted a genus-wide evolutionary analysis of the GAS8-domain protein, focusing on the *Trypanosoma melophagium* ortholog LSM04 004638. Maximum likelihood phylogenetic reconstruction, branch-specific and site-level codon-based selection analyses, ancestral sequence reconstruction, and Rosetta-based energetic estimation were performed. Despite strong purifying constraint consistent with essential cytoskeletal function, branch-level aBSREL detected a statistically significant signal compatible with episodic diversifying selection along the *T. melophagium* lineage (aBSREL: LRT = 6.72, Holm-corrected p = 0.0494). Site-level analyses identified two significant MEME codon sites (2 and 109; p = 0.00598 and p = 0.00916), corresponding to derived non-synonymous substitutions relative to the reconstructed ancestor (P2G and V106L); a third site (267) was suggestive but not significant (p = 0.08596). Branch-site codeml did not reach statistical significance (LRT = 1.7428, p = 0.1868, BH-FDR = 0.9339), although BEB highlighted site 2 (PP = 0.983) under the alternative model. These results are consistent with, but do not by themselves prove, limited lineage-specific adaptive refinement in an otherwise highly constrained cytoskeletal regulator; the signal should be regarded as method-dependent and statistically modest based on codeml branch-site test. A RELAX sensitivity test found no evidence that the focal-lineage signal is driven by relaxed constraint (K = 1.12, p = 0.697). Rosetta-based energetic comparisons across single- and double-reversion models consistently yielded positive $\Delta \Delta G$ values, but structural interpretation remains model-dependent and should be treated as hypothesis-generating rather than as direct evidence for altered function in vivo.

## Introduction

Trypanosomes are kinetoplastid parasites distinguished by a highly organized cytoskeletal architecture and a single motile flagellum, both of which are essential for cellular morphogenesis, environmental sensing, and host interaction. The subpellicular microtubule corset forms a cross-linked array underlying the plasma membrane and defines cell shape, polarity, and structural integrity. The flagellum, anchored via the basal body and flagellar pocket, drives motility and plays critical roles in parasite transmission, tissue tropism, and host–parasite interactions. Axonemal motility in eukaryotes depends on coordinated dynein-driven microtubule sliding regulated by multiprotein complexes associated with outer doublet microtubules. Among these regulators, the Growth arrest-specific protein 8 (GAS8) domain has emerged as a conserved structural module associated with dynein regulatory complexes and axonemal organization. In diverse eukaryotic systems, GAS8 homologs contribute to modulation of flagellar beating and mechanical control of motility. Within the genus *Trypanosoma*, Orthogroup 6127at5690 encodes a GAS8-domain-containing protein that is strictly single-copy in the validated analysis set. The *Trypanosoma cruzi* ortholog (C3747 40g126), annotated as a T-lymphocyte triggering factor, further suggests potential functional diversity within a structurally conserved framework. Gene Ontology annotations across species consistently indicate microtubule binding, cytoskeletal localization, and involvement in cell motility, supporting a conserved role in microtubule-associated architecture. The evolutionary profile of this orthogroup is striking. It is present in all surveyed *Trypanosoma* species, exhibits strict single-copy retention, shows minimal protein length variation (median 453 aa, ± 8.8 aa), and maintains intronless gene architecture. Such features are characteristic of essential housekeeping genes under strong purifying selection. Structural cytoskeletal proteins typically display high evolutionary constraint due to the necessity of preserving macromolecular interactions and mechanical integrity. However, strong overall constraint does not preclude localized adaptive modification. Even structurally indispensable proteins can accommodate limited residue-level changes that fine-tune protein–protein interactions, conformational flexibility, or mechanical properties. Ecological specialization, host specificity, and transmission biology may impose selective pressures that operate within conserved structural frameworks. *Trypanosoma melophagium*, a sheep-associated parasite transmitted by the sheep ked, occupies a distinct ecological niche compared to human- or livestock-infective relatives such as *T. brucei* or *T. cruzi*. Differences in host environment and transmission ecology may subtly influence requirements for motility efficiency or cytoskeletal resilience, creating opportunities for lineage-specific adaptive refinement, although such ecological interpretations remain hypotheses in the absence of functional validation. Codon-based models of molecular evolution provide statistical frameworks to detect such adaptive signals^1^. Branch-level models such as adaptive Branch-Site Random Effects Likelihood (aBSREL) detect episodic diversifying selection along specific lineages^2,3^, while site-level methods such as MEME identify codon positions subject to episodic positive selection across a subset of branches^4^. Integration of these approaches with ancestral sequence reconstruction allows identification of derived substitutions and evaluation of lineage-specific remodeling within conserved proteins. In this study, we investigate the evolutionary dynamics of the GAS8-domain protein LSM04 004638 in *Trypanosoma melophagium* within the broader context of Orthogroup 6127at5690. By combining phylogenetic reconstruction, codon-based selection analyses, ancestral inference, and energetic modeling of derived substitutions, we evaluate whether the available computational evidence is consistent with possible adaptive refinement within this otherwise highly conserved, single-copy cytoskeletal gene family.

## Methods

###  Sequence retrieval and ortholog identification

The GAS8-domain protein LSM04 004638 from *Trypanosoma melophagium* was selected as the focal sequence for evolutionary analyses. To delineate the corresponding orthologous group across the genus *Trypanosoma*, the *Trypanosoma cruzi* ortholog C3747 40g126—annotated as a T-lymphocyte triggering factor and associated with microtubule-binding functions—was retrieved from TriTrypDB (release 59 context in^5^) and used as the initial query for ortholog identification. Orthology assignments were initially inferred using a reciprocal BLASTP workflow across publicly available *Trypanosoma* proteomes. Putative one-to-one orthologs were required to satisfy reciprocal best-hit criteria under an E-value threshold of $1 \times 10^{-5}$ and high alignment coverage. To further validate ortholog relationships and exclude potential hidden paralogs, candidate sequences were cross-verified using OrthoDB annotations from the 2025 update release^6^. Only sequences consistently assigned to the same orthologous cluster in both RBH analysis and OrthoDB were retained for downstream evolutionary analyses. To avoid paralog interference in downstream codon-based selection analyses, only single-copy genes were retained. Gene copy number was verified by confirming the absence of additional high-scoring hits within each genome. Orthologs were further validated for conserved domain architecture using InterProScan, ensuring the presence of the Growth arrest-specific protein 8 (GAS8) domain (InterPro entries IPR025593 and IPR039308). Sequences lacking a complete GAS8 domain or exhibiting substantial truncation were excluded. All 10 unique *Trypanosoma* species represented in the TriTrypDB dataset used in this study were screened for the focal orthogroup. Because codon-based evolutionary analyses are sensitive to hidden paralogy, annotation error, and CDS disruption, final inclusion required: (i) reciprocal-best-hit support, (ii) concordant orthology assignment, (iii) single-copy status within each genome, (iv) preservation of the GAS8-domain architecture, and (v) an intact CDS lacking frameshifts or premature stop codons. Each of the 10 screened species satisfied these criteria, and the final codon-based analysis therefore comprised the complete validated *Trypanosoma* panel available within this study resource, rather than an arbitrarily reduced subset.

### Multiple sequence alignment and codon alignment construction

Protein sequences were aligned using PRANK^7^, a phylogeny-aware alignment algorithm that accounts for insertion–deletion events and reduces false-positive alignment errors. The resulting amino-acid alignment was manually inspected to ensure positional consistency. Codon alignments were generated from the protein alignment using PAL2NAL^8^, preserving reading-frame integrity and codon structure. To minimize alignment-induced artifacts in selection analyses, ambiguously aligned or gap-rich regions were removed using ClipKIT under the smart-gap mode^9^. For the final 10-taxon analysis set, ClipKIT reduced the protein alignment from 483 to 457 amino-acid sites (26 sites removed; 5.38%), yielding a 1371-nt codon alignment used in all downstream analyses.

### Phylogenetic reconstruction

Maximum likelihood (ML) phylogenetic reconstruction was performed using IQ-TREE v3.0.1^10^. The best-fitting amino-acid substitution model was selected using ModelFinder under the Bayesian Information Criterion (BIC)^11^. The Q.INSECT+F+G4 model was identified as optimal. Branch support was evaluated using 1000 ultrafast bootstrap replicates and 1000 SH-aLRT replicates. The resulting topology was used as a fixed reference phylogeny for codon-based molecular evolutionary analyses.

### Detection of branch-level episodic diversifying selection

Branch-specific episodic selection was evaluated using the adaptive Branch-Site Random Effects Likelihood (aBSREL) model implemented in HyPhy^2,3^. The trimmed codon alignment and ML phylogeny were provided as input. The MG94*×*REV codon substitution model was used as baseline. For each tested branch, the null hypothesis that all codon sites evolved under *ω* *łe* 1 was evaluated using a likelihood ratio test (LRT). To account for multiple testing across branches, p-values were adjusted using the Holm–Bonferroni correction^12^. Corrected p < 0.05 was considered statistically significant.

### Branch-site model analysis (codeml)

To independently validate branch-level signals, a branch-site model was implemented in codeml from PAML v4.10.9^1^. The *T. melophagium* lineage was specified as the foreground branch. Model A (allowing *ω> 1* on foreground branches) was compared against the corresponding null model (fix omega = 1, *ω* = 1 fixed) using a likelihood ratio test with one degree of freedom (df = 1). Positively selected codon sites were identified using Bayes Empirical Bayes (BEB) posterior probabilities. Sites with posterior probability *\ge* 0.95 were considered candidate sites conditional on the alternative model and were interpreted only in the context of the overall likelihood-ratio test result.

### Site-level episodic diversifying selection

Site-level episodic diversifying selection was assessed using MEME (Mixed Effects Model of Evolution) implemented in HyPhy^4^. MEME detects codon sites evolving under episodic positive selection on a subset of branches. Likelihood ratio tests were used to evaluate statistical significance, with p < 0.05 considered indicative of episodic diversifying selection.

###  Mapping of selected codon sites to extant and ancestral sequences

Codon sites identified under MEME were initially defined relative to the trimmed, gapped codon alignment. To map these positions to the ungapped extant LSM04 004638 protein sequence and to the reconstructed ancestral sequence, a custom Python script was implemented. The script tracked alignment gaps and restored original residue numbering in the extant protein. Corresponding ancestral residues were retrieved from maximum likelihood ancestral reconstruction output. Only sites with unambiguous one-to-one positional correspondence between trimmed alignment, extant sequence, and ancestral reconstruction were retained for interpretation.

### Ancestral sequence reconstruction

Ancestral states were reconstructed under maximum likelihood using codeml (PAML v4.10.9) with RateAncestor = 1, model = 0, NSsites = 0, and CodonFreq = 7 on the rooted ML topology. The internal node ancestral to the *T. melophagium*/*T. theileri* pair (Node #18) was translated for downstream residue mapping. Posterior probabilities were used to identify high-confidence ancestral states, with values *\ge* 0.95 considered strong support. Derived substitutions along the *T. melophagium* lineage were identified by comparing reconstructed ancestral states with the extant LSM04 004638 sequence.

### Energetic impact of derived substitutions

To evaluate structural contributions of lineage-specific substitutions, three ancestral-state reversion constructs were analyzed: G2P, L106V, and the combined double reversion (G2P + L106V). Input structures were generated from primary amino-acid FASTA sequences using AlphaFold Server (^13^; job metadata: dialect = alphafoldserver, version = 1). For each sequence, five candidate models were generated; the top-ranked model (model 0, highest ranking score) was used as the Rosetta input structure. Rosetta FastRelax^14^ simulations were performed with the default full-atom score function in PyRosetta (get fa scorefxn()) and no coordinate constraints. Ten independent replicate relaxations were used for each comparison (G2P, L106V, and double reversion). Total Rosetta Energy Units (REU) were recorded for each replicate. For the extant model, AlphaFold confidence values encoded in the B-factor column were summarized (mean 87.38, median 91.64, range 21.35–98.33; 3377/3725 atoms *\ge* 70). Confidence at focal residues was 62.17 (site 2) and 94.83 (site 106). Structural similarity between WT and mutant inputs was quantified using C*α* RMSD after Kabsch superposition. Stability differences were calculated as:

$$\begin{aligned} \Delta \Delta G = G_{\textrm{mutant}} - G_{\mathrm {wild\text{- }type}} \end{aligned}$$

Positive $\Delta \Delta G$ values were interpreted as model-inferred destabilization relative to the extant sequence. Mean and standard deviation were calculated across replicates. Because Rosetta $\Delta \Delta G$ estimates are sensitive to input structural models, relaxation settings, sampling depth, and score-function assumptions, they were treated as relative, hypothesis-generating indicators rather than direct evidence for altered protein stability or in vivo function.

### Statistical analysis

Likelihood ratio tests (LRTs) were used to evaluate model comparisons in both aBSREL and branch-site codeml analyses. For branch-site codeml, statistical significance was assessed using a chi-square distribution with one degree of freedom (df = 1). For aBSREL analyses, per-branch p-values were adjusted for multiple testing using the Holm–Bonferroni procedure. Corrected p < 0.05 was considered statistically significant. In MEME analyses, site-level significance was evaluated using likelihood ratio tests with p < 0.05 as the threshold. Bayes Empirical Bayes posterior probabilities *\ge* 0.95 were interpreted as strong support for positive selection at individual codon sites. All statistical tests were two-tailed. As all inferences were likelihood-based, no assumptions of normality were required. Additional robustness analyses were performed to test sensitivity of branch-level inference. First, RELAX^15^ was run with *T. melophagium* as the test branch and all remaining terminal *Trypanosoma* branches as reference. Second, aBSREL and MEME were repeated on an alternate 10-taxon codon alignment (458 codons; no Bodo outgroup) with an independently re-inferred ML tree.

## Results

### Genus-wide characterization of Orthogroup 6127at5690

Orthogroup 6127at5690 encodes a Growth arrest-specific protein 8 (GAS8) domain-containing protein conserved across the analyzed *Trypanosoma* dataset. The family was identified in 10/10 retained species and remained strictly single-copy in all included genomes. Protein length was highly conserved (median = 453 amino acids; SD = 8.8 aa). Gene architecture was uniformly intronless. Functional annotation consistently supported a cytoskeletal role, including microtubule binding (GO:0008017), involvement in cell motility (GO:0048870), and localization to microtubule-associated compartments (GO:0005856, GO:0005874). The absence of lineage-specific expansion and limited length variability indicate strong purifying selection at the genus level.

### Phylogenetic reconstruction

Phylogenetic relationships were inferred from an amino-acid alignment of 10 GAS8-domain orthologs comprising 457 sites. Of these, 110 sites (24.07%) were constant and 147 were parsimony-informative, distributed across 330 distinct site patterns. Maximum likelihood (ML) inference was conducted in IQ-TREE v3.0.1 using ModelFinder for substitution model selection under the Bayesian Information Criterion (BIC). ModelFinder identified Q.INSECT+F+G4 as the best-fit model (logL = −4307.869; BIC = 8842.351; BIC weight = 0.755). The model incorporates empirical amino-acid exchangeabilities optimized for insect proteins, observed state frequencies (+F), and among-site rate heterogeneity modeled using a discrete Gamma distribution with four rate categories (shape parameter *α* = 0.7977). The final ML tree had a log-likelihood of −4307.8568 with a total tree length of 4.5721 substitutions per site. Branch support was assessed using 1000 ultrafast bootstrap (UFBoot2) replicates and SH-like approximate likelihood ratio tests (SH-aLRT). Major nodes were strongly supported (e.g., SH-aLRT/UFBoot = 100/100 for the *T. vivax* + stercorarian clade; 100/100 for the *T. theileri* + *T. melophagium* sister relationship). The *Trypanosoma melophagium* sequence (LSM04 004638) formed a well-supported sister pair with *T. theileri* (100/100), consistent with their placement within the stercorarian lineage. This clade was nested within a broader assemblage including *T. grayi*, *T. congolense*, *T. rangeli*, and *T. cruzi*, although some internal relationships within this group received moderate support (e.g., 22.2/50 and 1.4/40 at deeper nodes), indicating limited phylogenetic resolution among certain taxa. The topology recovered was consistent between the ML tree and the extended consensus tree derived from bootstrap replicates (Robinson–Foulds distance = 2), indicating overall topological stability. Although the tree was inferred as unrooted, *T. brucei* was visualized as the outgroup for interpretative purposes. This ML topology was subsequently used as the fixed reference tree for all downstream codon-based selection analyses.

### Branch-level episodic diversifying selection

**Fig. 1:**
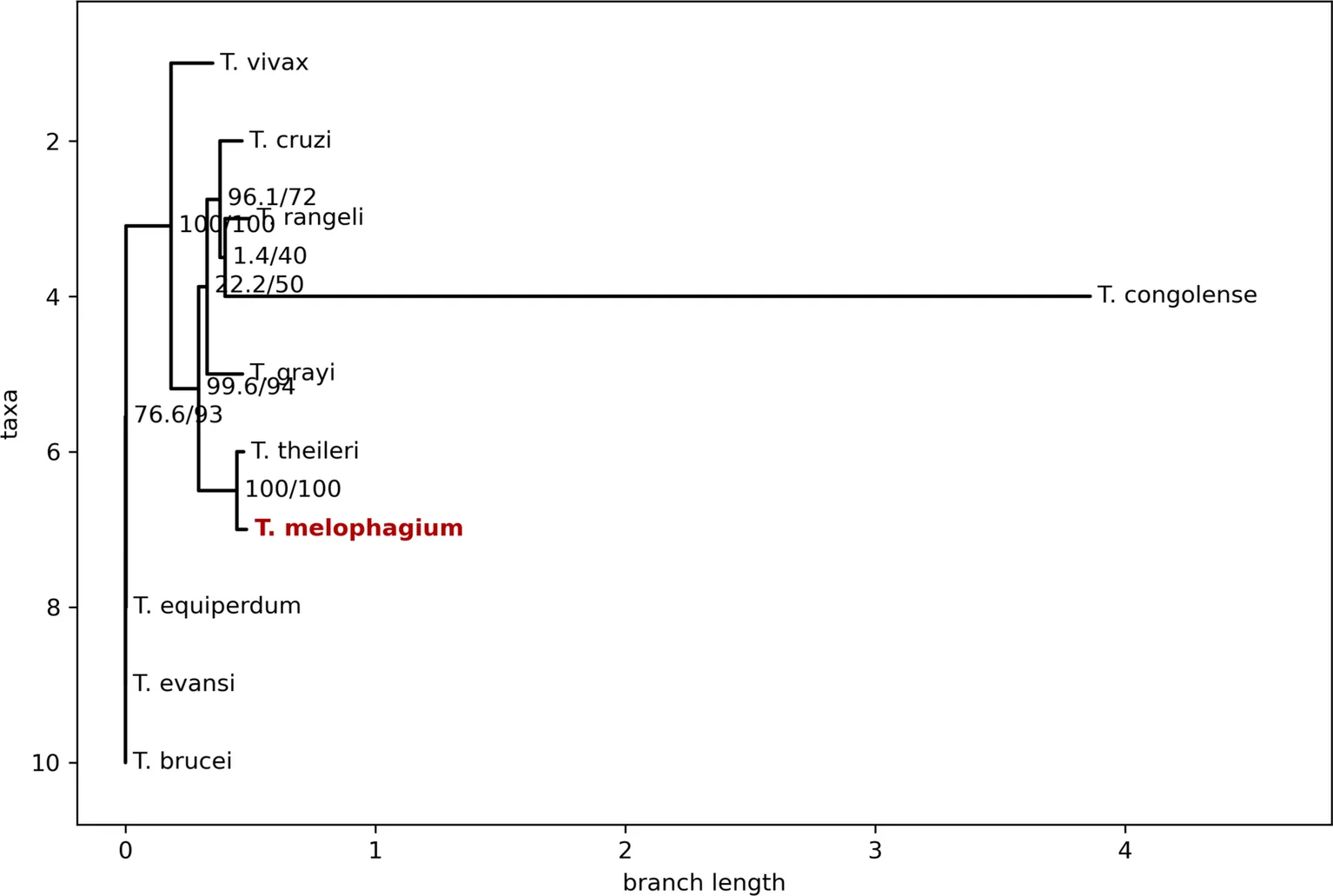
Maximum-likelihood phylogeny of GAS8-domain orthologs in the 10-*Trypanosoma*-taxon dataset used for codon-based analyses. The focal lineage, *T. melophagium*, is highlighted in red in the rendered figure.

aBSREL identified a statistically significant branch-level signal compatible with episodic diversifying selection on the *T. melophagium* lineage (LRT = 6.72; uncorrected p = 0.012; Holm–Bonferroni corrected p = 0.0494). Two *ω* categories were inferred on this branch. Approximately 98.1% of sites evolved under purifying or neutral selection (*ω*1 = 0.263), whereas 1.9% were assigned to a class with *ω*2 = 587.19, indicating a model-inferred high-*ω* category affecting a small subset of codons, a pattern compatible with episodic positive selection but not by itself sufficient for mechanistic inference. No additional branches remained significant after correction (Fig. 1).

###  Branch-site confirmation and site-level selection

Branch-site analysis using codeml did not detect statistically significant evidence of positive selection along the *T. melophagium* lineage. The likelihood ratio test comparing the alternative and null models yielded LRT = 1.7428 (p = 0.187), which was not significant after multiple-testing correction (BH-FDR = 0.934). Bayes Empirical Bayes (BEB) analysis identified a single codon (site 2) with posterior probability PP = 0.983, indicating elevated support under the alternative model. However, given the non-significant LRT, this site cannot be interpreted as robust evidence for lineage-specific positive selection. Collectively, these results suggest no statistically supported branch-site adaptive signal in the *T. melophagium* lineage under the codeml framework. MEME detected two significant codon sites under episodic diversifying selection in the trimmed alignment: positions 2 and 109. Site 267 showed suggestive but non-significant support.

### Mapping of selected sites

Mapping of trimmed alignment coordinates to the ungapped extant and ancestral sequences yielded the following correspondence:

**Table 1:** Mapping of MEME-evaluated codon sites from the trimmed alignment to extant and reconstructed ancestral residues in LSM04_004638.

Trimmed Site	Extant Position	Extant Residue	Ancestral Residue	Derived Change	MEME LRT	MEME *p*
2	2	G	P	P*→*G	8.6028	0.00598
109	106	L	V	V*→*L	7.7635	0.00916
267	264	Y	Y	Conserved	3.4129	0.08596

**Fig. 2:**
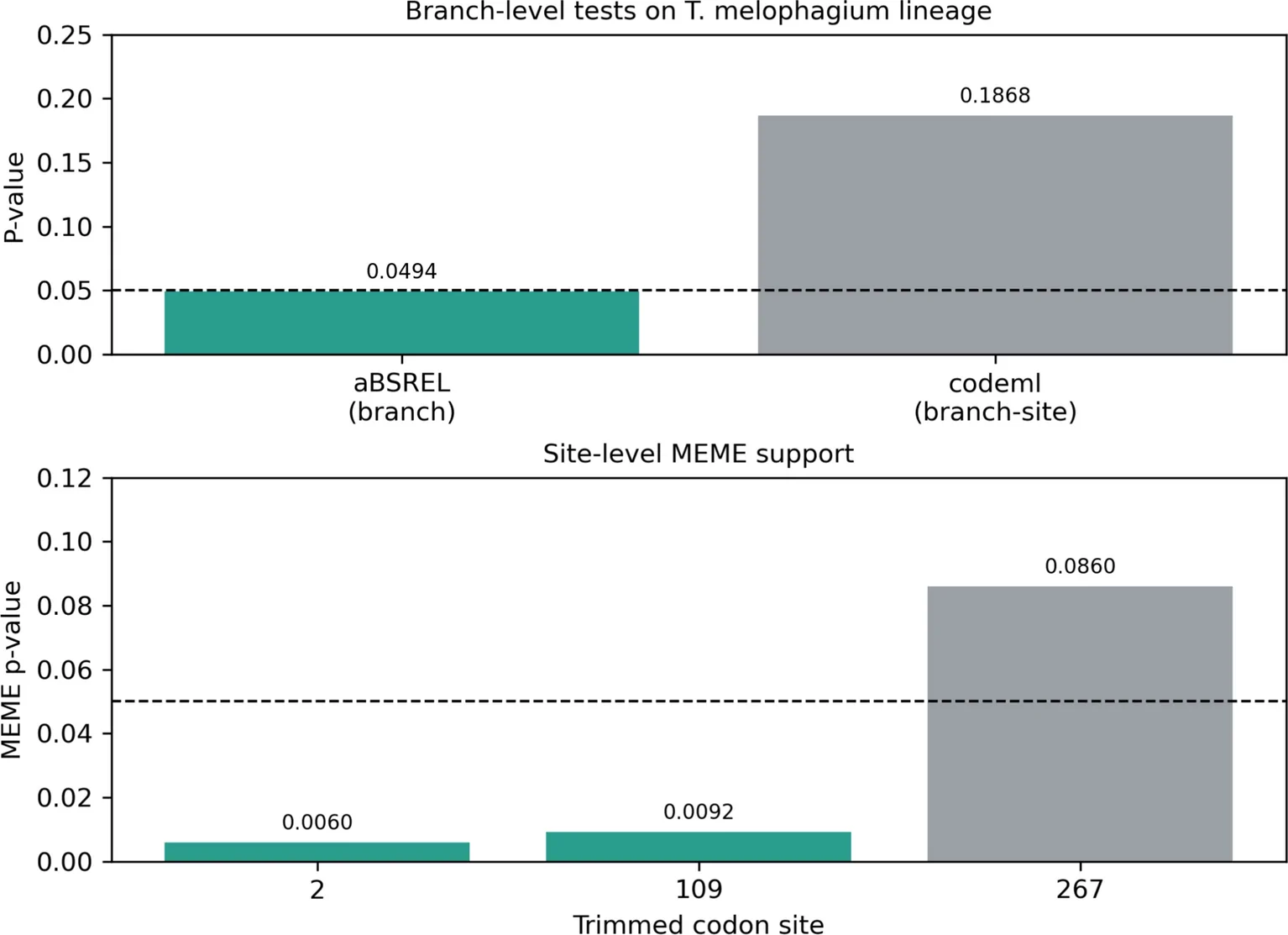
Summary of selection-test support levels. Top: branch-level tests on the *T. melophagium* lineage (aBSREL significant after Holm correction; branch-site codeml non-significant). Bottom: site-level MEME *p*-values for sites 2, 109, and 267. Dashed line marks *p = 0.05*.

The two significant MEME sites correspond to lineage-specific non-synonymous substitutions (P2G and V106L). Both substitutions lie N-terminal to the annotated canonical GAS8 domain (216–414), which limits direct mechanistic interpretation from domain architecture alone. Site 267 remained conserved in the extant lineage and did not pass the p < 0.05 threshold (Fig. 2).

###  Robustness analyses of selection signal

RELAX did not detect significant relaxation or intensification on the *T. melophagium* branch relative to the remaining *Trypanosoma* branches (K = 1.123, LRT = 0.152, p = 0.697), arguing against relaxed constraint as the primary explanation for the branch-level signal. In an alternate 10-taxon codon alignment (458 codons) with independently re-estimated topology, aBSREL still supported episodic diversification on the *T. melophagium* branch (LRT = 7.56, p = 0.00803). MEME on this alternate alignment retained site 2 as significant (LRT = 10.40, p = 0.00240), while additional sites reached significance, indicating that site-level calls are more alignment-sensitive than branch-level aBSREL support (Tables 1,2).

### Energetic consequences of derived substitutions

To resolve individual versus combined effects of derived substitutions, we evaluated single-site reversions (G2P and L106V) and the double reversion (G2P + L106V) relative to the extant WT model. All three comparisons yielded positive mean $\Delta \Delta G$ values (Table 3), consistent with destabilization of ancestral-state reversions in the modeled context. The L106V single reversion had the largest destabilizing effect (118.95 ± 14.05 REU), G2P showed a smaller but still positive effect (45.49 ± 17.38 REU), and the double reversion was intermediate (67.44 ± 7.35 REU), indicating non-additive behavior. WT-versus-mutant C*α* RMSD remained high globally (double: 114.59 Å; G2P: 37.59 Å; L106V: 105.62 Å), with substantially lower local RMSD in focal windows (1–20 and 90–120), consistent with limited local perturbation superimposed on broader model-level rearrangement. Because Rosetta energies are model-dependent internal scores, we interpret this pattern as hypothesis-generating and not as direct evidence for altered protein function in vivo.

## Discussion

Orthogroup 6127at5690 represents a conserved, single-copy cytoskeletal gene family across the genus *Trypanosoma*. Universal presence, strict single-copy retention, limited protein length variation, and intronless architecture^5^ collectively indicate strong long-term purifying selection and essential cellular function. Functional annotations consistently support a microtubule-associated role. Enrichment for microtubule binding, cytoskeletal localization, and cell motility aligns with the established involvement of GAS8 homologs in dynein regulatory complexes and axonemal organization^5^. In kinetoplastids, where morphology and motility are tightly coupled to

**Table 2:** Summary of molecular evolution analyses evaluating lineage-specific selection signals in GAS8-domain protein LSM04 004638 of *Trypanosoma melophagium*.

Analysis	Key Result	Statistical Support	Interpretation
*aBSREL*	*T. melophagium* branch under episodic selection	LRT = 6.72; corrected *p* = 0.0494; $\omega _2$ = 587.19 (1.9% of sites)	Branch-specific episodic selection
Branch-site codeml	No significant branch-site support on the focal lineage	LRT = 1.7428; *p* = 0.1868; BH-FDR = 0.9339; BEB: site 2, PP = 0.983	Suggestive site-level signal under ALT model, but not significant overall
MEME	Sites 2 and 109 significant; site 267 suggestive	Site 2: LRT = 8.6028, *p* = 0.00598; Site 109: LRT = 7.7635, *p* = 0.00916; Site 267: *p* = 0.08596	Episodic diversification at specific residues with mixed support strength

**Table 3:** Rosetta $\Delta \Delta G$ summary for ancestral-state reversion models relative to extant WT *T. melophagium* LSM04_004638. Positive values indicate destabilization.

Comparison	Replicates	Mean $\Delta \Delta G$ (REU)	Range (REU)
Single G2P reversion	10	45.486 ± 17.381	7.508 to 65.782
Single L106V reversion	10	118.954 ± 14.051	97.284 to 138.018
Double G2P+L106V reversion	10	67.439 ± 7.348	53.630 to 76.584

subpellicular microtubule organization and flagellar mechanics, preservation of structural integrity is critical. Despite strong genus-wide constraint, branch-level analyses provided method-dependent evidence compatible with episodic diversifying selection along the *T. melophagium* lineage. The adaptive signal was restricted to a small proportion of codon sites, consistent with localized refinement rather than widespread structural change. Such a pattern is expected for essential structural proteins, which rarely tolerate extensive amino-acid variation but may undergo limited adjustments affecting regulatory interactions or mechanical properties. Site-level analyses identified two significant codon positions (2 and 109), both corresponding to lineage-specific non-synonymous substitutions (P2G and V106L). A third site (267) showed only suggestive support. Extant positions 2 and 106 are N-terminal to the annotated GAS8 domain (216–414), indicating residue-level remodeling outside the core annotated domain. In the structural model, site 2 also falls in a lower-confidence region than site 106, further supporting conservative interpretation. Branch-site codeml analysis did not independently support positive selection on the focal lineage (LRT = 1.7428, p = 0.1868). Although BEB highlighted site 2 (PP = 0.983) under the alternative model, this should be interpreted cautiously given the non-significant model comparison. This non-significant codeml result is important because it shows that the adaptive signal is not uniformly supported across methods; instead, the evidence depends primarily on aBSREL and MEME and should be considered statistically modest. A complementary RELAX analysis found no evidence that the focal branch is dominated by relaxed selective constraint (K = 1.123, p = 0.697). Energetic analysis of single and double reversions showed consistent positive $\Delta \Delta G$ values, indicating model-inferred destabilization when ancestral residues were reintroduced into the present modeled background. The larger effect for L106V reversion versus G2P, and the intermediate double-reversion effect, supports a non-additive energetic pattern in the Rosetta model space. These findings are compatible with context-dependent stabilizing contributions of the derived residues, but they do not establish a mechanism. Because the comparison is based on predicted structures and Rosetta internal units, and because Rosetta $\Delta \Delta G$ estimates are sensitive to model assumptions, relaxation protocol, and conformational sampling, the magnitude should not be interpreted as a direct physical free-energy estimate or as evidence for altered function in vivo; experimental validation remains necessary. The ecological specialization of *T. melophagium* may plausibly provide selective context. Differences in host environment and transmission biology could impose subtle demands on motility or cytoskeletal resilience, potentially promoting adaptive fine-tuning within a conserved framework. However, alternative explanations, including stochastic lineage-specific drift or compensatory change within a strongly constrained protein background, cannot be excluded from the present computational data. Collectively, these findings illustrate how essential cytoskeletal regulators can remain highly conserved across deep evolutionary timescales while accommodating limited lineage-specific residue-level change. In this system, possible adaptive refinement is supported primarily by branch-level aBSREL evidence and MEME site-level results, with mixed support across complementary methods and no statistically significant codeml branch-site confirmation. Taken together, the present computational evidence should be viewed within clear inferential limits. While codon-based models provide statistical evidence compatible with episodic diversification, the signal is method-dependent and remains modest, particularly because the codeml branch-site test was non-significant. Functional consequences of identified substitutions remain to be experimentally validated, and their location outside the canonical GAS8 domain limits direct mechanistic inference. Structural and motility assays will be required to determine whether the inferred changes affect protein interactions or flagellar dynamics; stochastic drift or compensatory evolution within a constrained protein background also remain plausible alternatives.

## Conclusion

The GAS8-domain protein LSM04 004638 in *Trypanosoma melophagium* is a highly conserved, single-copy cytoskeletal regulator that nonetheless shows a limited, method-dependent signal consistent with possible lineage-specific adaptive refinement. Codon-based analyses identify two derived substitutions outside the canonical GAS8 domain along the *T. melophagium* lineage, while branch-level and site-level methods provide partially concordant but modest support. These results suggest the hypothesis that adaptive evolution within essential cytoskeletal proteins may proceed through targeted residue-level optimization without disrupting core structural architecture. Structural interpretation from Rosetta remains hypothesis-generating and should be validated experimentally.

## Supplementary Information

Supplementary Information.

## Data Availability

The datasets generated and analyzed during the current study are available in the Zenodo repository, https://doi.org/10.5281/zenodo.20428385.

## References

[1] Yang, Z. PAML 4: Phylogenetic analysis by maximum likelihood. *Mol. Biol. Evol.*10.1093/molbev/msm088 (2007). 17483113 10.1093/molbev/msm088

[2] Smith, M. D. et al. Less is more: An adaptive branch-site random effects model for efficient detection of episodic diversifying selection. *Mol. Biol. Evol.* **32**(5), 1342–1353 (2015). 25697341 10.1093/molbev/msv022 PMC4408413

[3] Pond, S. L. K. et al. HyPhy 2.5-a customizable platform for evolutionary hypothesis testing using phylogenies. *Mol. Biol. Evol.* **37**(1), 295–299 (2020). 31504749 10.1093/molbev/msz197 PMC8204705

[4] Murrell, Ben et al. Detecting individual sites subject to episodic diversifying selection. *PLoS Genetics* **8**(7), e1002764 (2012). 22807683 10.1371/journal.pgen.1002764 PMC3395634

[5] Shanmugasundram, Achchuthan et al. TriTrypDB: An integrated functional genomics resource for Kinetoplastida. *PLoS Neglected Tropical Diseases* **17**(1), e0011058 (2023). 36656904 10.1371/journal.pntd.0011058 PMC9888696

[6] Tegenfeldt, F. et al. OrthoDB and BUSCO update: Annotation of orthologs with wider sampling of genomes. *Nucleic Acids Res.* **53**(D1), D516–D525 (2025). 39535043 10.1093/nar/gkae987 PMC11701741

[7] Löytynoja, A. Phylogeny-aware alignment with PRANK. *Methods Mol. Biol.* **2231**, 155–170 (2022). 10.1007/978-1-62703-646-7_10 24170401

[8] Suyama, M., Torrents, D. & Bork, P. PAL2NAL: Robust conversion of protein sequence alignments into the corresponding codon alignments. *Nucleic Acids Res.* **34**(Web Server issue), W609–W612 (2006). 16845082 10.1093/nar/gkl315 PMC1538804

[9] Steenwyk, Jacob L. et al. ClipKIT: a multiple sequence alignment-trimming algorithm for accurate phylogenomic inference. *PLoS Biology* **18**(12), e3001007 (2020). 33264284 10.1371/journal.pbio.3001007 PMC7735675

[10] Minh, B. Q. et al. IQ-TREE 2: New models and efficient methods for phylogenetic inference in the genomic era. *Mol. Biol. Evol.* **37**(5), 1530–1534 (2020). 32011700 10.1093/molbev/msaa015 PMC7182206

[11] Kalyaanamoorthy, S., Minh, B. Q., Wong, T. K. F., von Haeseler, A. & Jermiin, L. S. ModelFinder: Fast model selection for accurate phylogenetic estimates. *Nat. Methods* **14**(6), 587–589 (2017). 28481363 10.1038/nmeth.4285 PMC5453245

[12] Aickin, M. & Gensler, H. Adjusting for multiple testing when reporting research results: The Bonferroni vs Holm methods. *Am. J. Public Health* **86**(5), 726–728 (1996). 8629727 10.2105/ajph.86.5.726 PMC1380484

[13] Jumper, John et al. Highly accurate protein structure prediction with AlphaFold. *Nature* **596**(7873), 583–589 (2021). 34265844 10.1038/s41586-021-03819-2 PMC8371605

[14] Sora, V. et al. RosettaDDGPrediction for high-throughput mutational scans: From stability to binding. *Protein Sci.* **32**(1), e4527 (2023). 36461907 10.1002/pro.4527 PMC9795540

[15] Wertheim, J. O., Murrell, B., Smith, M. D., Pond, S. L. K. & Scheffler, K. RELAX: Detecting relaxed selection in a phylogenetic framework. *Mol. Biol. Evol.* **32**(3), 820–832 (2015). 25540451 10.1093/molbev/msu400 PMC4327161

